# Engineered Bacterial Membranes as Next‐Generation Platforms for Cancer Immunotherapy

**DOI:** 10.1002/smsc.70351

**Published:** 2026-07-26

**Authors:** Hafiza Aasia Malik, Urooj Yousaf Virk, Mohamed Salah Attia, Jenny Wilson, Ming Wei

**Affiliations:** ^1^ School of Pharmacy and Medical Sciences (PAM) Griffith University Gold Coast Queensland Australia

**Keywords:** bacterial ghosts (BGs), bacterial membrane‐based vaccines, cancer immunotherapy, immune modulation, nanovesicle engineering, neoantigen vaccines, outer membrane vesicles (OMVs), tumor microenvironment

## Abstract

Bacterial membranes, in their natural and engineered forms, including outer membrane vesicles, bacterial ghosts, engineered membrane fragments, and hybrid scaffolds, are emerging as multifunctional immunotherapeutic platforms that merge antigen presentation with intrinsic adjuvanticity. By codisplaying tumor antigens and conserved pathogen‐associated molecular patterns (PAMPs) such as lipopolysaccharide, flagellin, and CpG motifs, bacterial membranes activate dendritic cells, drive crosspresentation, and elicit durable cytotoxic T‐cell memory. Advances in genetic fusion systems (Lpp‐OmpA, ClyA, Ag43, and SpyTag/SpyCatcher), lipid A detoxification, and tumor membrane hybridization have transformed bacterial membranes from empirical immunostimulants into programmable vaccine scaffolds. Preclinical studies across melanoma, lung, breast, and glioblastoma models show that these systems reprogram the tumor microenvironment, inducing Th1‐polarized immunity, pyroptotic tumor death, and synergy with checkpoint blockade, chemotherapy, and phototherapy. Beyond vesicular formats, membrane fragments and engineered ghosts demonstrate equivalent potential for safe, modular, and scalable vaccine design. Integrating AI‐driven antigen discovery, CRISPR‐based strain engineering, and automated biofoundries now offers a path toward clinical translation. Collectively, these developments position bacterial membranes as a unifying platform that bridges innate and adaptive immunity for next‐generation cancer immunotherapy.

## Graphical abstract

Engineered bacterial membrane platforms, including outer membrane vesicles, bacterial ghosts, genetically engineered membranes, and hybrid bacterial–tumor membranes, integrate tumor antigen presentation with intrinsic immune stimulation. These platforms promote dendritic‐cell activation, T‐cell priming, pyroptosis, and synergistic responses with checkpoint blockade, ultimately driving tumor regression and durable antitumor immunity.

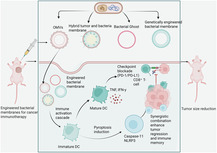

## 1 Introduction

**FIGURE 1:**
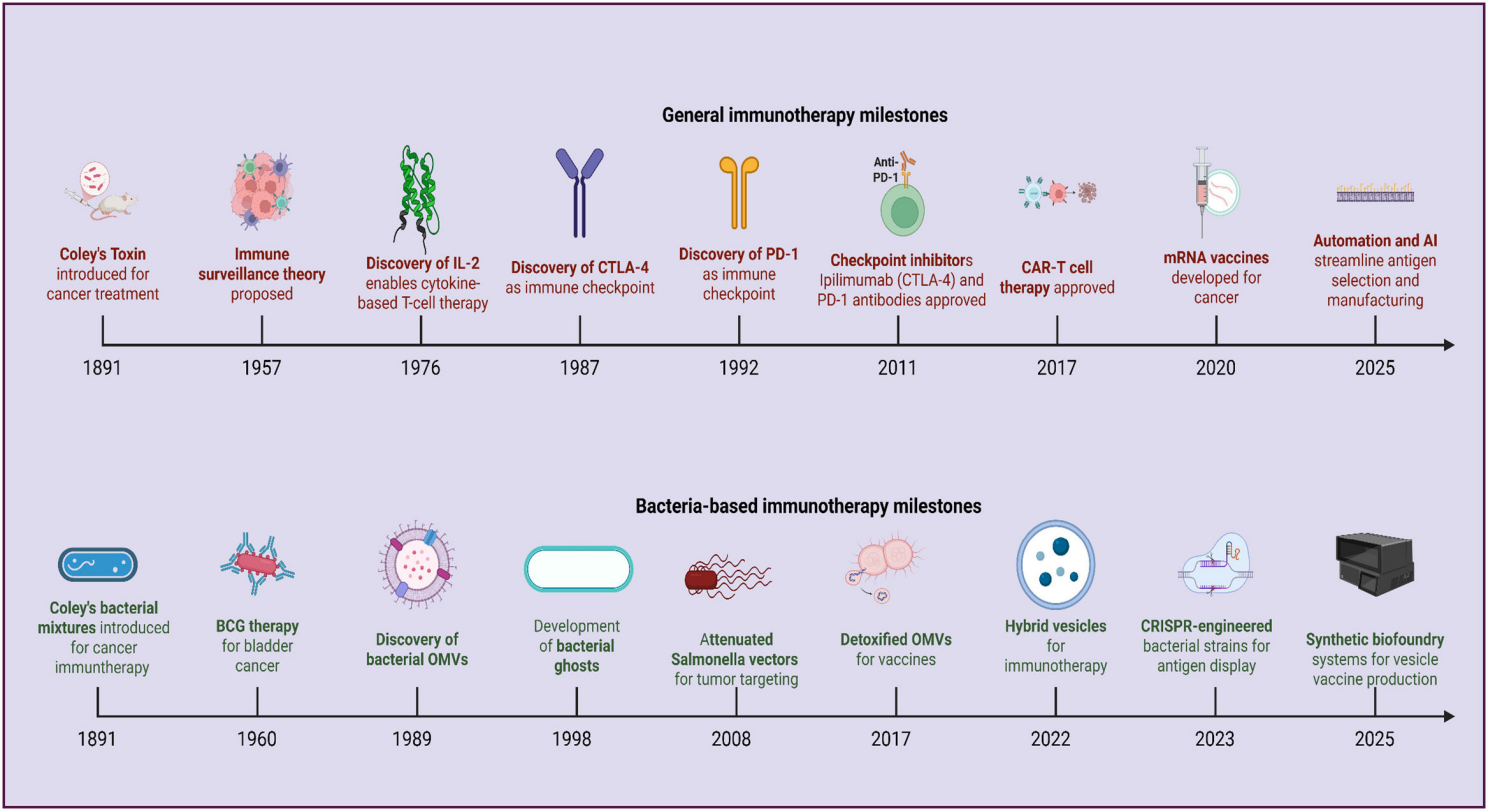
Historical evolution of cancer immunotherapy and bacteria‐based vaccine platforms.

Harnessing the immune system to combat cancer has undergone a profound evolution, moving from empirical use for infections to highly developed cellular and molecular strategies. In the late 19^th^ century, William Coley laid the foundation for cancer immunotherapy by introducing “Coley’s toxins,” showing that bacterial components could activate immune responses capable of targeting tumors [1, 2, 3]. A few decades later, Paul Ehrlich proposed the concept of immunosurveillance, suggesting that the immune system acts as a natural guardian, detecting and eliminating emerging cancer cells before they become dangerous [4, 5]. Eventually, this idea evolved into the broader theory of immunoediting, which describes how the immune system shapes tumor growth and protects against it. During the sequential phases of elimination, equilibrium, and escape, tumor cells are first eliminated by immune surveillance, subsequently maintained in a state of immune‐mediated control, and eventually acquire adaptations that enable immune evasion, including reduced antigen presentation, increased immune checkpoint signaling, and the accumulation of immunosuppressive cell populations [1, 6, 7, 8]. These discoveries eventually led to the development of modern immunotherapy, where immune checkpoint inhibitors such as CTLA‐4 (cytotoxic T‐lymphocyte‐associated protein 4) and PD‐1 (programmed cell death protein 1) blockade have transformed the treatment of cancers like melanoma, lung, and renal carcinomas by stimulating exhausted T‐cells [1, 3, 9]. Another breakthrough is the ex vivo reprogramming of T‐lymphocytes, which yielded chimeric antigen receptor T (CAR‐T) cell therapy with long‐lasting responses, particularly in hematologic malignancies (Figure 1) [3].

Despite these advances, current cancer vaccines still face significant limitations. Vaccines based on viral vectors induce potent immunity but carry risks of pre‐existing antivector immunity and inflammatory toxicity [3, 7]. Similarly, vaccines based on peptides and proteins are simple to manufacture but suffer from restricted epitope breadth, weak immunogenicity, and the need for strong adjuvants [1, 10]. Following their success in the COVID‐19 pandemic, mRNA vaccines have become leading candidates for cancer immunotherapy owing to rapid design, multivalent antigen encoding, and intrinsic immunostimulatory potential [10, 11, 12]. Delivered mRNA directs antigen expression through MHC I and II pathways to activate CD8^+^ and CD4^+^ T cells [13]. Yet molecular instability, endosomal entrapment, and type I interferon (IFN‐I) responses limit translation and durability, particularly within heterogeneous, immunosuppressive tumors [14, 15, 16, 17].

While these molecular platforms have advanced tumor immunotherapy, they rely on synthetic carriers that lack innate immunostimulatory signals. In contrast, bacterial membranes inherently integrate both antigen scaffolding and adjuvanticity within a single biological structure [18]. Bacterial outer membranes are inherently enriched with pathogen‐associated molecular patterns (PAMPs), such as lipopolysaccharide (LPS), flagellin, and unmethylated CpG motifs. These PAMPs are known to engage Toll‐like receptors (TLRs) (e.g., TLR4, TLR5, TLR9) and nucleotide‐binding oligomerization domain (NOD)‐like receptors to trigger proinflammatory cytokine release and dendritic cell maturation [1, 3]. This intrinsic adjuvanticity distinguishes bacterial membranes from synthetic delivery systems such as lipid nanoparticles used in mRNA vaccines. While mRNA vaccines rely on intracellular translation of encoded antigens following cellular uptake, bacterial membrane vesicles simultaneously deliver tumor antigens and PAMPs within a single biological structure [19]. The colocalization of tumor antigens and PAMPs promotes coordinated activation of antigen‐presenting cells (APCs) and adaptive immune responses without the need for separate adjuvant formulations [20]. In addition, bacterial membrane platforms support multivalent antigen display and membrane engineering strategies that enable integration of tumor‐derived components and immunomodulatory cargos within the same nanoscale scaffold, thereby enhancing antigen presentation and T‐cell priming [7]. For instance, fusion systems such as the lipoprotein‐outer‐membrane protein chimera (Lpp‐OmpA), originally developed for antigen surface display in *E. coli*, have been widely exploited across vaccine studies [21, 22]. Parallel strategies to attenuate endotoxicity, such as deletion of *msbB* (encoding lipid A lauroyltransferase) or *htrB* (encoding lipid A myristoyltransferase) to yield hypo‐acylated lipid A variants, demonstrate how rational engineering can enhance both safety and immunogenicity [23]. Nevertheless, excessive activation of innate immune pathways may result in systemic inflammatory responses, including fever and cytokine release, emphasizing the need to balance vaccine potency with safety during clinical translation.

This review aims to provide an integrated perspective on how bacterial cell membranes, in their diverse engineered forms, including natural OMVs, synthetic hybrids, and rationally modified membrane scaffolds, are being harnessed for cancer immunotherapy. While previous reviews have often focused narrowly on OMVs in infectious disease vaccines or discussed bacterial ghosts (BGs) in isolation, here we specifically highlight the application of bacterial membranes in oncology. We outline recent engineering strategies encompassing antigen display, lipid A detoxification, conjugation, tumor membrane hybridization, and functional payload delivery and compare these approaches with established vaccine modalities. In doing so, this review highlights bacterial membranes as uniquely versatile scaffolds that merge evolutionary adjuvanticity with synthetic programmability for next‐generation cancer vaccines.

## 2 Bacterial Membranes as Vaccine Platforms

Bacterial membrane‐derived systems, ranging from naturally released vesicles and bacterial ghosts to synthetic and hybrid constructs, have emerged as promising vaccine platforms that combine inherent immunostimulatory properties with the ability to present tumor antigens for effective cancer immunotherapy.

### 2.1 Naturally Derived Bacterial Vesicles for Antigen Delivery and Immune Activation

In recent years, bacterial membrane‐based systems have become increasingly popular due to the need for improved vaccine platforms. Bacterial OMVs are nanosized blebs (20–250 nm) released by Gram‐negative bacteria during growth or stress [24]. *Escherichia coli‐*derived OMVs have been shown to activate cytotoxic T lymphocytes through interferon‐mediated pathways [25]. OMVs bring their own adjuvant properties while also serving as highly adaptable scaffolds for antigen display. The intrinsic adjuvanticity of bacterial membranes arises from membrane‐associated PAMPs that engage multiple pattern‐recognition receptors on antigen‐presenting cells. Lipid A, the immunologically active component of lipopolysaccharide (LPS), activates TLR4, triggering NF‐κB and IFN‐I signaling pathways that promote proinflammatory cytokine production and dendritic‐cell maturation. Similarly, flagellin activates TLR5, while unmethylated CpG DNA stimulates TLR9, further enhancing innate and adaptive immune responses. In addition, peptidoglycan fragments and outer membrane proteins can activate NOD‐like receptor pathways, reinforcing innate immune signaling. Together, these pathways promote upregulation of costimulatory molecules, facilitate antigen crosspresentation through major histocompatibility complex class I pathways, and support the priming and expansion of tumor‐specific CD8^+^ T cells. Beyond their molecular cargo, the physicochemical properties of bacterial membrane vesicles influence their biological behavior. Parameters such as particle size, surface charge, membrane composition, and fluidity affect biodistribution, lymphatic trafficking, and cellular uptake. OMVs can access lymphoid tissues and are internalized by antigen‐presenting cells through multiple pathways, including clathrin‐mediated endocytosis, caveolin‐dependent uptake, and membrane fusion, thereby influencing antigen processing, crosspresentation, and the magnitude of downstream immune responses [26, 27, 28].

In modular “plug‐and‐display” OMV systems, dual‐antigen presentation has been shown to elicit potent T‐cell immunity, significantly reducing pulmonary metastases and enhancing IFN‐γ‐producing CD8^+^ and CD4^+^ T‐cell responses [29]. Using a SpyCatcher‐SpyTag‐based plug‐and‐display approach, OMVs carrying the neoantigen Adpgk (adenosine diphosphate–dependent glucokinase) induced durable tumor suppression with high survival rates and complete regression in most treated mice [30]. Mechanistically, these OMVs promoted marked intratumoral infiltration of CD8^+^ and CD4^+^ T cells, DCs, and activated neutrophils while alleviating the immunosuppressive influence of regulatory T cells. Similarly, OMVs displaying *human papillomavirus type 16* (HPV‐16) E7 antigens achieved enhanced surface expression and generated robust antigen‐specific CD8^+^ T‐cell responses, leading to effective tumor regression and lymphocyte recruitment in the TC‐1 lung cancer model [31]. Collectively, these findings underscore the therapeutic potential of OMV‐based vaccines, which not only inhibit metastatic progression but also remodel the tumor microenvironment to sustain long‐term immune control.

**FIGURE 2:**
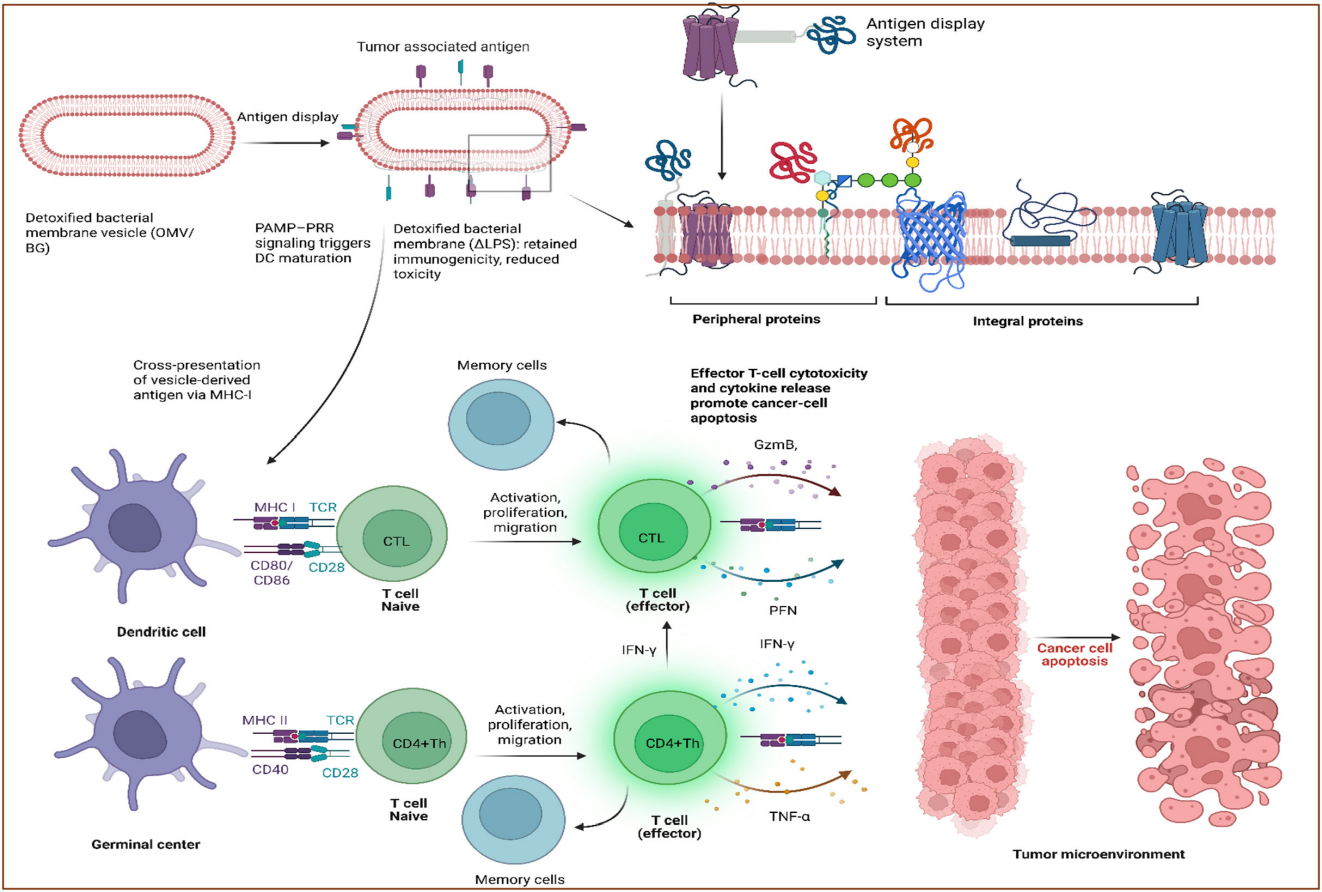
Detoxified bacterial membrane vesicles (OMVs/BGs) displaying tumor‐associated antigens engage pattern‐recognition receptors (PAMP–PRR) on dendritic cells (DCs), triggering maturation and crosspresentation via MHC I and II. Activated DCs prime naïve CD8^+^ cytotoxic (CTL) and CD4^+^ helper (Th) T cells, leading to effector differentiation, cytokine secretion (IFN‐γ, TNF‐α), and cytolytic activity (perforin, granzyme B). These coordinated responses induce cancer‐cell apoptosis and establish memory T‐cell immunity within the tumor microenvironment.

In parallel, BGs, empty bacterial envelopes generated through controlled lysis, preserve native surface antigens and membrane integrity while lacking genetic material. Their intrinsic adjuvanticity, structural stability, and compatibility with therapeutic coloading make them attractive scaffolds for cancer immunotherapy. BGs hybridized with tumor membranes and combined with chemotherapeutics have demonstrated synergistic antitumor activity, reducing tumor burden and enhancing CD4^+^/CD8^+^ T‐cell activation and proinflammatory cytokine production (Figure 2) [32]. Together, OMVs and BGs exemplify naturally derived bacterial membrane platforms capable of multivalent antigen presentation, potent CD8^+^ T‐cell priming, and measurable tumor control in vivo [30, 31].

### 2.2 Synthetic and Hybrid Bacterial‐Tumor System

#### 2.2.1 Bacterial‐Tumor Membrane Hybrids

Hybrid vesicles formed by fusing bacterial and tumor membranes combine bacterial adjuvanticity with tumor antigenicity, creating a unified platform for simultaneous immune stimulation and tumor‐specific recognition [33]. reported the generation of hybrid nanocarriers through fusion of *E. coli* cytoplasmic membranes with autologous tumor cell membranes, which codelivered PAMPs and tumor antigens. These hybrids effectively activated DCs, expanded CD8^+^ T‐cell populations, and prevented postoperative tumor recurrence in murine models. Such constructs exemplify how membrane fusion can endow vaccines with both innate and adaptive immunogenic features that are difficult to achieve using conventional formulations [33].

#### 2.2.2 Membrane‐Coated and Multifunctional Synthetic Vesicles

Synthetic strategies extend hybrid design by coating bacterial vesicles with tumor membranes or incorporating therapeutic components to enhance efficacy. Tumor‐membrane‐cloaked OMVs retain bacterial immunostimulatory cues while acquiring tumor tropism and partial immune evasion, thereby improving antigen delivery and lymphocyte activation [34]. Likewise, cancer‐cell‐membrane‐coated BGs preserve tumor antigenicity and deliver bacterial danger signals, amplifying antigen recall responses and increasing splenic CD4^+^/CD8^+^ T‐cell activation and cytokine production [32].

Recent multifunctional designs have broadened these concepts. You et al. [34] developed OMVs coloaded with doxorubicin and CD47‐siRNA and modified with Angiopep‐2 to facilitate blood‐brain‐barrier penetration in glioblastoma models. These vesicles induced immunogenic cell death, reprogrammed macrophages and microglia toward prophagocytic phenotypes, and markedly prolonged survival, illustrating the adaptability of bacterial membranes for complex therapeutic applications.

Bai et al. [35] reviewed how OMVs and hybrid bacterial membranes improve vaccine efficacy by enabling multivalent antigen loading, adjuvant‐antigen colocalization, and crosstalk with the tumor microenvironment, resulting in enhanced T‐cell responses and improved tumor control in preclinical models.

Collectively, these studies highlight the versatility of membrane‐coated and multifunctional bacterial vesicles as modular platforms capable of integrating targeting ligands, chemotherapeutics, nucleic acids, and tumor‐derived antigens within a single construct. Such multifunctional designs enable simultaneous modulation of innate immunity, antigen presentation, and tumor‐specific targeting, thereby expanding the therapeutic potential of bacterial membrane‐based immunotherapies [35]. To facilitate comparison among currently available membrane‐based platforms, Table 1 summarizes the key biological characteristics, engineering features, advantages, and limitations of bacterial membrane systems relative to mammalian exosomes.

**TABLE 1:** Comparison of bacterial membrane‐based platforms and mammalian exosomes for cancer immunotherapy.

Feature	OMVs	Bacterial ghosts (BGs)	Hybrid bacterial membranes	Mammalian exosomes	References
**Biological origin**	Naturally shed from Gram‐negative bacterial outer membranes	Empty bacterial envelopes generated after removal of cytoplasmic contents	Fusion of bacterial membranes or OMVs with mammalian/tumor membranes	Endosome‐derived extracellular vesicles secreted by mammalian cells	[35, 36]
**Typical size**	20–250 nm	Retains parent bacterial morphology (micron‐scale)	Variable; depends on OMV/membrane fusion method	∼30–150 nm	[24, 35, 36, 37, 38]
**Main composition**	Bacterial lipids, outer‐membrane proteins, periplasmic cargo, LPS, and other PAMPs	Preserved bacterial envelope with surface antigens and PAMPs, but lacking viable cytoplasmic content	Combined bacterial immune‐stimulatory components and mammalian/tumor membrane antigens	Lipid bilayer vesicles containing proteins, lipids, metabolites, DNA/RNA, and cell‐derived surface proteins	[20, 35]
**Detoxification strategy**	Lipid A engineering (*lpxL*, *lpxM*, *pagP*), PEGylation	Removal of intracellular contents	Combination with mammalian membranes, lipid modification	Generally, not required	[39, 40, 41, 42]
**Intrinsic adjuvanticity**	High, due to bacterial PAMPs	High, due to retained bacterial envelope components	Tunable; bacterial component provides immune stimulation; tumor membrane broadens antigenic repertoire	Generally low unless derived from immune cells or specifically engineered	[43, 44, 45]
**PAMP content**	Present	Present	Present but variable depending on bacterial membrane proportion	Absent in conventional mammalian exosomes	[1, 2, 3, 7, 20]
**Antigen presentation potential**	Supports surface or luminal antigen loading; can be genetically engineered for antigen display	Can retain native surface antigens and be loaded with tumor antigens or drugs	Can present both bacterial danger signals and tumor‐derived antigens	Can carry tumor antigens, peptide‐MHC complexes, or engineered cargo depending on donor cell/source	[14, 20]
**Cargo‐loading strategies**	Genetic engineering, membrane display, electroporation, incubation, extrusion or postisolation conjugation	Loading through electroporation, incubation or membrane association	Coextrusion, sonication, membrane fusion, nanoparticle coating or drug encapsulation	Passive incubation, donor‐cell loading, sonication, extrusion, electroporation, click chemistry or surface modification	[36]
**Immunogenicity**	Strong; useful for vaccine immune activation but may cause excessive inflammation	Strong; nonreplicating but still immunostimulatory	Adjustable; aims to balance bacterial immunogenicity with tumor targeting or immune evasion	Lower basal immunogenicity; useful for biocompatible delivery but usually requires engineering for strong immune activation	[12, 23, 46, 47]

**Safety concerns**	Endotoxin‐related toxicity, systemic cytokine responses and batch heterogeneity	Residual bacterial components and inflammatory responses	More complex safety profile due to mixed membrane composition and manufacturing variability	Donor‐cell heterogeneity, unwanted cargo transfer, purification challenges and variable biodistribution	[35, 48]
**Tumor‐targeting potential**	Requires engineering or ligand modification	Requires engineering or tumor‐membrane coating	Enhanced when tumor membranes provide homotypic targeting	May show natural or engineered tropism depending on donor cell and surface modification	[14]
**Manufacturing scalability**	Scalable through bacterial culture, but requires endotoxin control and quality standardization	Scalable bacterial production, but requires controlled lysis and purification	More complex due to membrane fusion and multicomponent quality control	More difficult to scale consistently because yield and properties depend on donor cell type and culture conditions	[35, 48]
**Key limitation**	Balancing adjuvanticity with toxicity	Maintaining safety while preserving immunogenicity	Reproducibility and regulatory complexity	Low intrinsic adjuvanticity and manufacturing heterogeneity	

#### 2.2.3 Nanoparticle‐Integrated and Advanced Hybrid Systems

**FIGURE 3:**
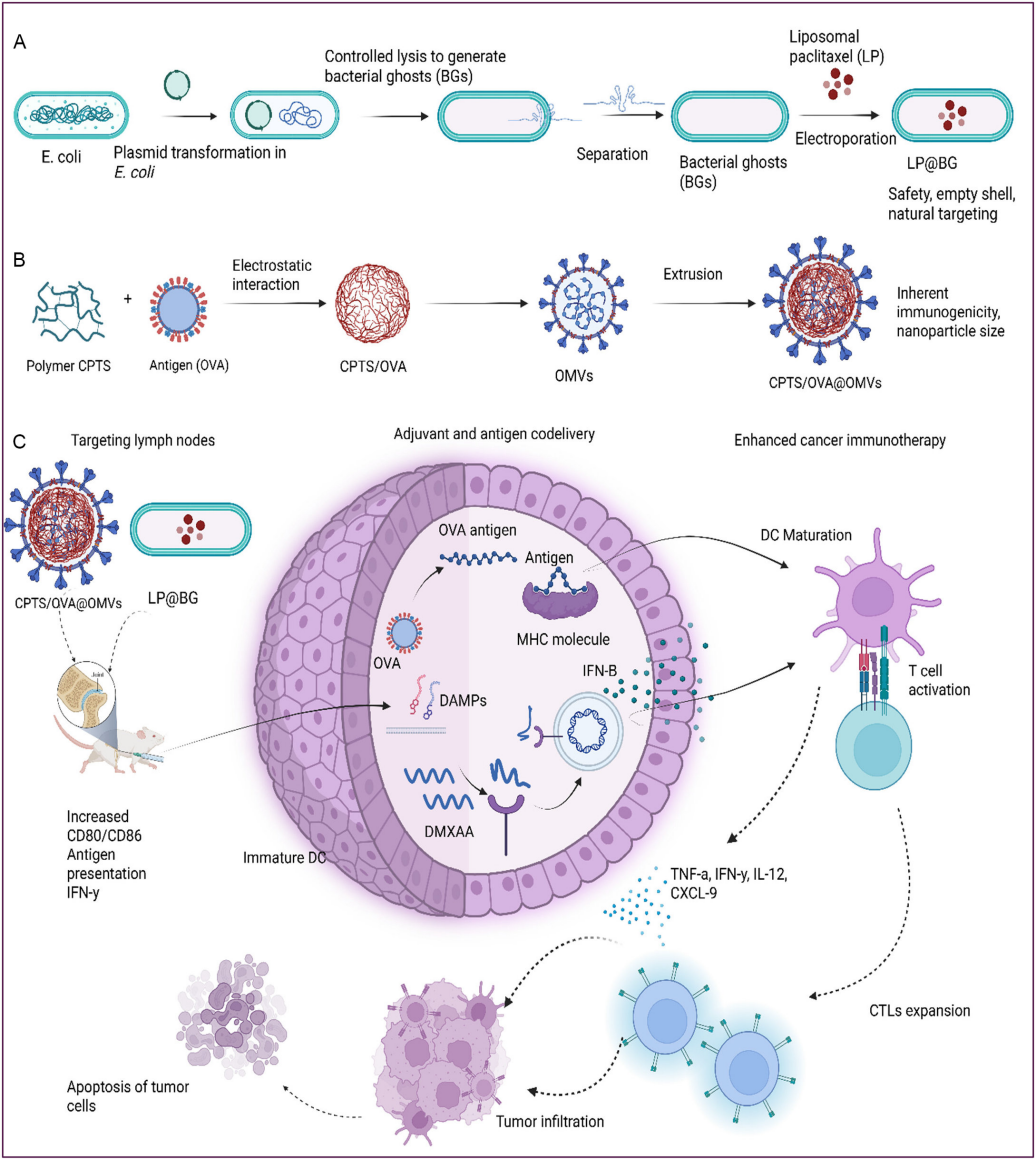
**Bacterial ghosts (BGs) and OMVs as complementary delivery systems for cancer immunotherapy.** (A) Controlled lysis of plasmid‐transformed *E. coli* generates bacterial ghosts (BGs), which are empty shells retaining membrane architecture. Liposomal paclitaxel [46] is introduced by electroporation, producing liposomal paclitaxel encapsulated within bacterial ghosts (LP@BG) constructs with safety, natural targeting, and efficient drug loading [16]. (B) Outer membrane vesicles (OMVs) were engineered through electrostatic incorporation of polymer–antigen complexes composed of poly(β‐amino ester)‐tethered ovalbumin (CPTS/OVA) followed by extrusion, generating CPTS/OVA@OMVs with inherent immunostimulatory properties and nanoscale delivery characteristics [16]. (C) Both LP@BG and CPTS/OVA@OMVs target lymph nodes, where they enhance dendritic‐cell [52] maturation, antigen presentation, and costimulatory signaling (CD80/CD86, IFN‐γ, IFN‐β). This cascade drives T‐cell activation, cytokine secretion (TNF‐α, IFN‐γ, IL‐12, CXCL‐9), CTL expansion, and tumor infiltration, culminating in apoptosis of tumor cells and improved antitumor immunity.

Integration of bacterial membranes with nanomaterials or additional membrane sources has yielded advanced constructs that strengthen both innate and adaptive immune responses. Lin et al. [49] synthesized OMV‐cloaked mesoporous silica nanoparticles carrying CpG oligonucleotides, which promoted extensive tumor regression, enhanced M1 macrophage polarization, DC maturation, and durable tumor‐specific immune memory in breast and colorectal cancer models. In related work, Yang and Wu [50] and Zou et al. [51] designed membrane‐hybrid tumor OMVs (mTOMVs) that increased intratumoral CD8^+^ T‐cell infiltration and suppressed immunosuppressive myeloid populations compared with native OMVs, demonstrating cooperative activation of innate and adaptive pathways [50, 51]. (Figure 3) provides an overview of the generation and functional mechanisms of these bacterial membrane‐derived vaccine platforms.

Collectively, bacterial membranes provide biologically evolved yet synthetically adaptable scaffolds that merge antigen presentation with self‐adjuvanticity. Their natural immunostimulatory capacity and engineering flexibility form the foundation for programmable vaccine platforms, the design principles of which are explored in the following section.

## 3 Engineering Strategies for Bacterial Membrane Vaccines

**FIGURE 4:**
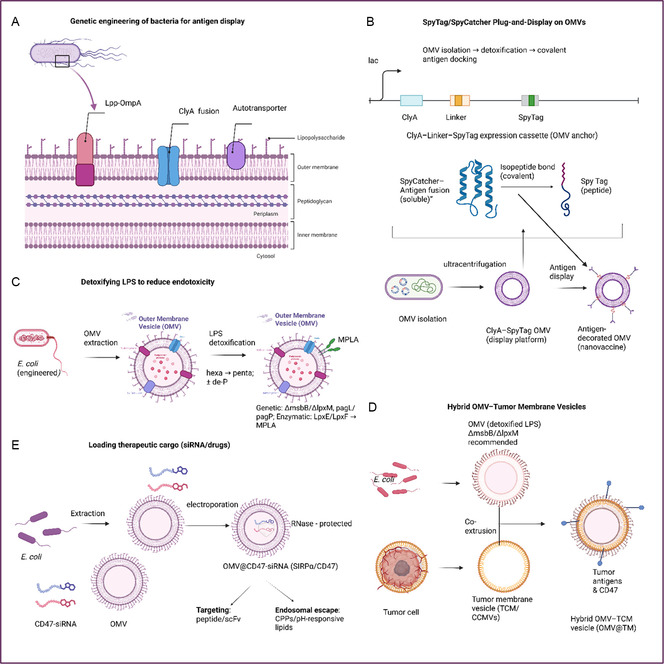
**Engineering strategies for bacterial outer membrane vesicles (OMVs) in cancer immunotherapy.** (A) Genetic engineering of *E. coli* enables antigen display on OMVs through fusion systems such as ClyA, autotransporters, or Lpp‐OmpA. A SpyTag fusion can be anchored on OMVs, allowing covalent attachment of SpyCatcher–antigen constructs via a “plug‐and‐display” strategy. (B) Schematic of OMV composition, showing outer membrane proteins, porins, and periplasmic contents that can be harnessed for functionalization. (C) OMVs are isolated by ultracentrifugation and subjected to LPS detoxification (e.g., ΔmsbB/ΔlpxM mutants) to reduce endotoxicity, producing safe vaccine platforms. (D) Hybrid vesicles can be generated by coextrusion of bacterial outer membrane vesicles (OMVs) with cancer‐cell membrane vesicles (CCMVs), yielding OMV@TM constructs that combine bacterial adjuvant properties with tumor‐derived antigens [16]. (E) OMVs can also be engineered as delivery vehicles for therapeutic cargos, such as siRNA (e.g., CD47‐siRNA), loaded by electroporation and protected from RNase degradation, with additional modifications for targeting and endosomal escape.

Bacterial membranes and their engineered derivatives offer a unique foundation for cancer vaccine design, functioning simultaneously as antigen carriers and intrinsic adjuvants through conserved PAMPs. To maximize their therapeutic efficacy, these systems require precise engineering to balance immunogenicity, antigen specificity, and safety. Recent advances in genetic display platforms, modular conjugation chemistries, endotoxin detoxification, and biomimetic membrane hybridization collectively enable the generation of next‐generation vesicular vaccines based on bacterial cell membranes with enhanced CD8^+^ T‐cell priming, superior tumor microenvironment reprogramming, and improved safety profiles relative to unmodified bacterial membranes (Figure 4). A comparison of bacterial membrane vaccines with established vaccine platforms, including mRNA, viral vector, and peptide vaccines, is provided in Table 2 to highlight their respective strengths and translational considerations.

**TABLE 2:** Contrasts bacterial membrane platforms with mRNA, viral, and peptide vaccines, highlighting antigen delivery, adjuvanticity, stability, safety, and production feasibility. Bacterial membranes uniquely combine antigen presentation with intrinsic immune stimulation and are compatible with modular engineering systems (e.g., Plug‐and‐Display, autotransporter fusions).

Feature	Bacterial membranes (OMVs, BGs)	mRNA vaccines	Viral vectors	Peptide vaccines
**Antigen delivery**	Direct surface display or hybrid fusion	Transient in vivo expression	Stable, but risk of vector immunity	Limited, requires adjuvants
**Adjuvanticity**	Strong intrinsic via PAMPs (TLR, NOD)	Requires adjuvants	Vector backbone provides some	Weak, needs potent adjuvant
**Stability**	Stable vesicles, lyophilizable	Cold chain required	Moderate; risk of recombination	Stable but low immunogenicity
**Safety**	Reduced by LPS detoxification, nonreplicating	Generally safe	Risk of insertional mutagenesis	Safe, but low efficacy
**Durability**	Induces robust memory T cells	Often short‐lived	Strong but limited by pre‐existing immunity	Weak durability
**Representative quantitative outcomes**	60% complete tumor regression; 70% survival; complete tumor rechallenge protection reported in preclinical models	Variable by platform	Variable by platform	Variable by formulation
**Production**	Scalable bacterial fermentation	Cell‐free in vitro transcription	Complex cell culture	Peptide synthesis scalable

### 3.1 Genetic Display Systems: Anchors and Secretion Pathways

Genetic fusion of tumor antigens to outer‐membrane scaffolds is an established strategy for surface display in bacterial membrane vaccines. Among the best‐characterized systems, (Lpp‐OmpA) chimera, originally developed in *E. coli*, has been widely adopted for stable antigen anchoring across vesicular and ghost platforms [21, 22]. These studies collectively demonstrate the versatility of Lpp‐OmpA in presenting heterologous proteins, including tumor‐associated fragments, while maintaining membrane stability.

The pore‐forming cytolysin (ClyA) represents another effective carrier, with *Salmonella* strains engineered to express ClyA showing efficacy in colorectal cancer but limited activity in breast cancer models. This limitation was overcome by replacing ClyA with Clostridium perfringens enterotoxin [53], which binds the tight‐junction protein CLDN4, commonly overexpressed in breast tumors [53, 54]. CPE expression enhanced infiltration of CD4^+^, CD8^+^, and natural killer (NK) nucleotide‐binding oligomerization domain cells while reducing neutrophil‐driven inflammation, underscoring the importance of matching bacterial carriers to tumor‐specific microenvironments.

Autotransporters such as antigen 43 (Ag43) and hemoglobin protease (Hbp) constitute another adaptable secretion system composed of a signal peptide, passenger domain, and β‐barrel translocator [55]. Substituting the passenger domain enables export and stable surface display of large or complex antigens [56, 57]. Hbp has supported multivalent presentation of tumor epitopes, while engineered Ag43 signal peptides have improved folding and transport efficiency, expanding the repertoire of complex cargos, including enzymatic fusions and viral receptor‐binding domains, that can be displayed [58, 59].

Collectively, these genetic systems demonstrate how membrane‐anchored antigen display has evolved from simple fusion constructs into flexible, modular tools for bacterial membrane‐based vaccine engineering.

### 3.2 Conjugation and Modular Assembly

Beyond genetic fusion, antigen conjugation strategies allow covalent attachment of epitopes or proteins onto bacterial vesicles, offering flexible routes for vaccine customization. Conventional chemical conjugation methods, including NHS‐ester crosslinkers (e.g., BS3), carbodiimide chemistry using 1‐ethyl‐3‐(3‐dimethylaminopropyl) carbodiimide (EDC), or bio‐orthogonal click reactions, enable direct coupling of peptides, proteins, or glycans to vesicle surfaces. These late‐stage functionalization approaches facilitate control of antigen density and allow incorporation of chemically synthesized tumor epitopes. For instance, OMVs decorated genetically with synthetic tumor epitopes have been shown to significantly boost dendritic‐cell activation and elicit antigen‐specific CD4^+^ and CD8^+^ T cell responses [60, 61].

By contrast, the SpyTag/SpyCatcher “plug‐and‐display” system represents a next‐generation conjugation strategy. This technology enables irreversible isopeptide bond formation between a short peptide (SpyTag) engineered onto the antigen and the SpyCatcher protein predisplayed on OMVs [62]. This system allows for “plug‐and‐display” assembly of vesicles with diverse tumor antigens, without the need to re‐engineer bacterial strains [63, 64]. It supports rapid assembly of vesicles with diverse tumor antigens without the need to re‐engineer bacterial strains, thereby offering unmatched modularity. In preclinical studies, this system accelerated antigen presentation and elicited strong T‐cell responses, underscoring its potential for patient‐tailored cancer vaccines [30].

### 3.3 Strategies for Balancing Endotoxicity and Adjuvanticity

Bacterial membranes carry the strong adjuvanticity innately, but their endotoxin activity needs to be carefully managed. Lipid A, the immunostimulatory component of LPS, drives TLR4 signaling intensity depending on its acylation state. Hexa‐acylated lipid A not only potently activates NF‐κB and proinflammatory cytokine release but also induces reactogenicity. Genetic modifications in *lpxL* (*lipid A myristoyltransferase*) or *lpxM* (*lipid A lauroyltransferase*) produce penta‐acylated variants with attenuated toxicity. A prominent application of this strategy is ClearColi, a detoxified *E. coli* chassis in which *lpxL*, *lpxM*, *pagP*, and *eptA* are deleted, forcing accumulation of Lipid IV_a_‐a nonendotoxic precursor that retains structural integrity for OMV production while exhibiting minimal TLR4 activation [39, 40]. Additional enzymatic modifications using *pagL* (lipid A deacylase) or *lpxR* (lipid A dephosphorylase) further trim or remodel lipid A to temper inflammation while maintaining sufficient TLR4 signaling [41, 42]. More recently, dynamic shielding approaches such as PEGylation or cloaking with host‐derived membranes have been developed to dampen systemic cytokine surges without compromising dendritic‐cell maturation [35]. These detoxified strains preserve the dual advantage of antigen display and intrinsic immune activation with minimal systemic toxicity. Importantly, attenuation of endotoxicity does not necessarily compromise antitumor efficacy. Detoxified OMVs have been shown to retain the capacity to activate dendritic cells, promote CD8^+^ T‐cell responses, and suppress tumor growth in preclinical models. For example, detoxified OMVs derived from the Δ60 strain inhibited melanoma progression, increased immune‐cell infiltration within the tumor microenvironment, and generated durable antitumor immunity capable of protecting against tumor rechallenge [65]. These findings suggest that rational lipid A engineering can improve safety while preserving the immune activation required for effective cancer vaccination.

### 3.4 Synthetic and Hybrid Bacterial‐Tumor Systems

Engineering efforts have extended beyond genetic modifications to create hybrid vesicles that integrate bacterial and tumor membrane components. Fusion of OMVs with autologous tumor cell membranes enables simultaneous delivery of bacterial PAMPs and tumor antigens, thereby combining potent innate cues with tumor specificity [33]. Building on this principle, cloaking strategies have coated OMVs or bacterial ghosts with tumor membranes to confer antigen repertoires while retaining bacterial danger signals. More advanced designs include paclitaxel‐loaded BGs enveloped with lung cancer membranes, which paired cytotoxic payload delivery with enhanced T‐cell activation [32]. reported paclitaxel‐loaded BGs cloaked with lung cancer membranes that suppressed metastasis, reduced lung weights from ∼813 mg to ∼328 mg (approaching ∼250 mg in healthy mice), and increased splenic CD4^+^/CD8^+^ T cells along with TNF‐α/IFN‐γ secretion [32], and OMV‐cloaked mesoporous silica nanoparticles carrying CpG oligonucleotides, which promoted macrophage polarization and durable tumor‐specific memory [49]. Collectively, these synthetic hybrids highlight a modular approach to uniting bacterial adjuvanticity with tumor‐derived targeting in one vesicle platform.

### 3.5 Functional Payload Integration

Beyond membrane fusion, bacterial vesicles have been engineered as carriers for therapeutic payloads. OMVs coloaded with doxorubicin and CD47‐siRNA and further decorated with the brain‐targeting peptide Angiopep‐2 exemplify multifunctional constructs that cross physiological barriers while delivering both cytotoxic and immune‐modulating cues [34]. Parallel approaches have embedded siRNAs targeting immune checkpoints such as PD‐L1 or nanobody fragments to amplify T‐cell responses and sustain tumor regression [35]. These multifunctional vesicles demonstrate the versatility of bacterial membranes as integrated carriers for antigens, adjuvants, and therapeutic agents.

Collectively, these engineering approaches, ranging from genetic anchoring and chemical conjugation to detoxification and hybridization, redefine bacterial membranes as programmable, self‐adjuvating vaccine platforms. Each strategy contributes to precise antigen display, immune‐response tuning, and enhanced translational potential, forming the mechanistic basis for their preclinical success discussed in the next section.

## 4 Preclinical and Emerging Clinical Evidence

### 4.1 Murine Tumor Models: Efficacy Trends Across Melanoma, Lung, Breast, and Colorectal Cancer

Murine models remain the foundation for evaluating bacterial membrane vaccines. In melanoma, the B16‐F10 model consistently demonstrates potent antitumor immunity. OMVs engineered to express model antigens such as OVA suppressed tumor growth, prolonged survival, and enhanced CD8^+^ T‐cell infiltration [30]. Intratumoral injection of detoxified OMVs (Δ60 strain) inhibited growth in up to 60% of mice and achieved complete responses in a subset [66]. Treated mice rejected tumor rechallenge, confirming durable immune memory. Mechanistically, OMV therapy promoted dendritic‐cell infiltration, CD80/CD86 upregulation, NK‐cell recruitment, and tumor necrosis within 24 h, accompanied by pyroptotic cell death via the caspase‐11/NLRP3 axis [66, 67].

In metastatic lung cancer, BGs coated with tumor membranes have shown superior therapeutic efficacy. Paclitaxel‐loaded BGs cloaked with autologous lung‐tumor membranes achieved homotypic targeting, enhanced uptake, and significantly reduced metastasis compared with free drug or uncoated BGs. Treated mice displayed elevated CD8^+^ T‐cell infiltration and reduced suppressive myeloid cells [32, 65]. The combined presence of bacterial PAMPs and tumor antigens produced a Th1‐polarized response characterized by IFN‐γ and TNF‐α induction [37].

Additional tumor contexts further underscore the versatility of bacterial membranes. In breast cancer, OMVs engineered to overexpress pre‐miRNA reduced 4T1 tumor growth compared with control vectors, supporting their use as RNA‐delivery vaccines [68]. In colorectal cancer, ClearColi‐derived OMVs displaying CT26 neoepitopes elicited potent CD8^+^ T‐cell responses, delayed tumor progression, and prolonged survival [43]. Moreover, oral delivery of engineered *E. coli* producing antigen‐bearing OMVs induced protective immunity in both metastatic melanoma and subcutaneous colon models. Across tumor types, these vaccines consistently elicit Th1‐dominant immunity, durable memory, and therapeutic benefit in models resistant to conventional vaccination. To bridge these murine findings with human relevance, bacterial membrane vaccines have also been explored in humanized and organoid systems.

### 4.2 Humanized Models and Organoid Systems

Humanized mouse studies confirm that OMVs carrying melanoma neoantigens expand human CD8^+^ T cells, elevate IFN‐γ, and reduce tumor burden [43]. Patient‐derived xenograft (PDX) systems incorporating both human tumor and immune compartments enable evaluation of bacterial vesicle‐based immunotherapies in clinically relevant settings [69, 70]. ClearColi OMVs encoding CT26 neoepitopes induced central memory T cells and delayed tumor progression in humanized colorectal models [43, 71]. Complementary results arise from organoid systems: in colorectal PDO‐PBMC cocultures, neoantigen OMVs elicited stronger cytotoxicity and Th1 cytokine release than peptide vaccines [65]. While in breast cancer patient‐derived organoids (PDOs), tumor‐cloaked BGs enhanced antigen‐specific lymphocyte infiltration and IFN‐γ secretion [37]. Reviews highlight that PDO platforms enable rapid, scalable testing of OMVs, BGs, and hybrid vesicles, accelerating personalized vaccine development [35].

### 4.3 Clinical Snapshots

Early clinical translation of personalized cancer vaccines in melanoma is underway: a first‐in‐human personalized neoantigen vaccine trial demonstrated safety and immunogenicity (NCT01970358). In parallel, outer membrane vesicle (OMV) platforms are advancing preclinically. A plug‐and‐display OMV‐mRNA system elicited potent CD8^+^ responses, significantly inhibited melanoma progression, and achieved complete regressions in a colon model in mice [72]. In situ intratumoral delivery of detoxified OMVs triggered tumor necrosis, DC and NK‐cell recruitment, systemic antitumor immunity, and durable rechallenge rejection in B16 and other models [66, 73].

**FIGURE 5:**
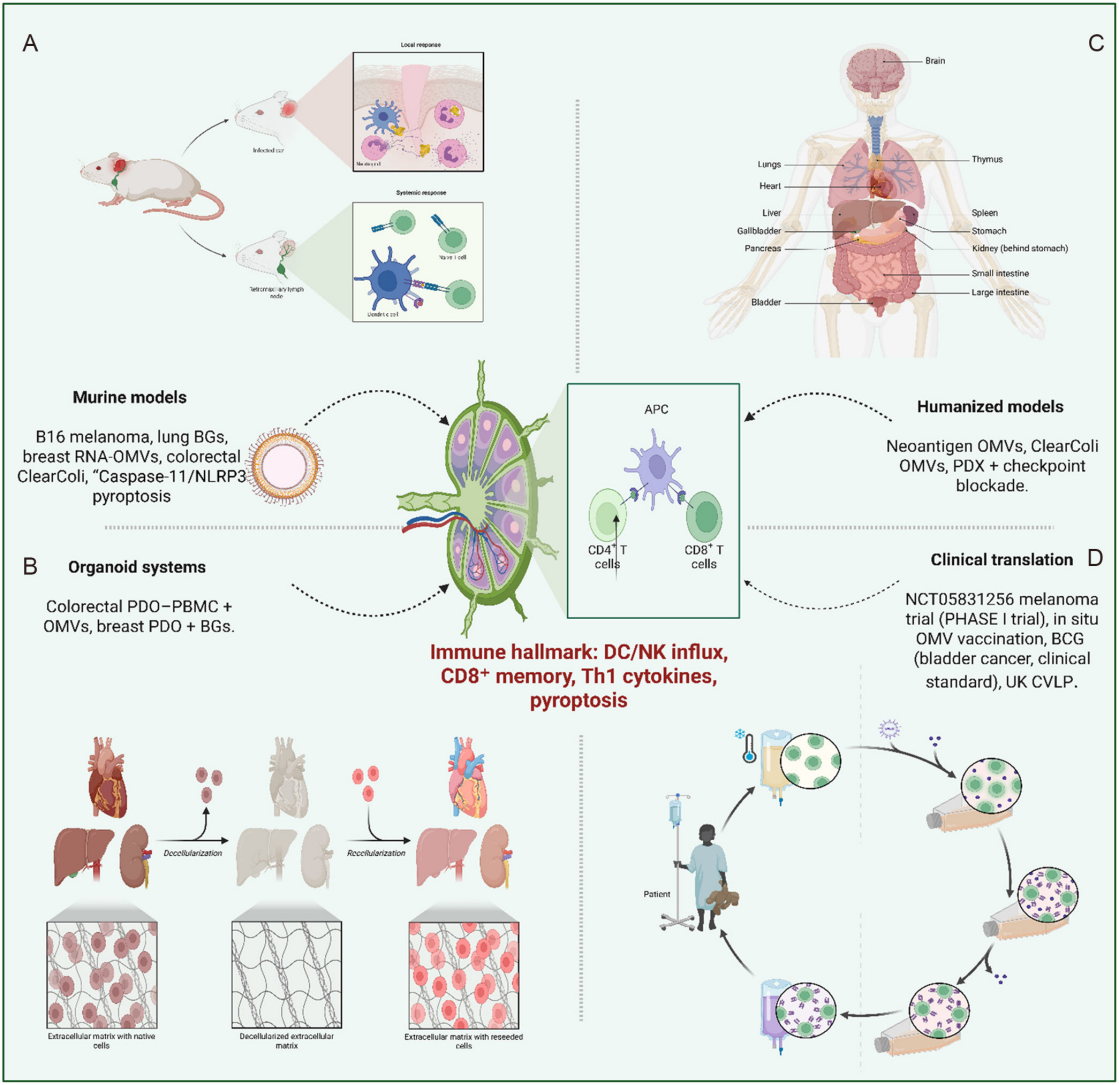
**Preclinical and emerging clinical evidence for bacterial membrane‐based cancer vaccines.** (A) Murine models: B16 melanoma, lung BGs, breast RNA‐OMVs, and colorectal ClearColi elicit robust antitumor immunity marked by DC infiltration, NK recruitment, and caspase‐11/NLRP3‐driven pyroptosis. (B) Organoid systems: Patient‐derived organoids cocultured with PBMCs reveal enhanced lymphocyte infiltration and Th1 cytokine release upon OMV or BG exposure. (C) Humanized models: Neoantigen and ClearColi OMVs, alone or with checkpoint blockade, expand human CD8^+^ T cells, elevate IFN‐γ, and suppress tumor growth. (D) Clinical translation: Early trials include a first‐in‐human OMV melanoma vaccine (NCT05831256) and in situ OMV immunotherapy, alongside established bacterial vaccines such as BCG. Immune hallmarks across models include DC/NK influx, Th1 polarization, CD8^+^ memory expansion, and pyroptosis‐driven crosspresentation.

Outside melanoma, Bacillus Calmette–Guérin (BCG) remains a standard of care for nonmuscle‐invasive bladder cancer [74]. National initiatives such as the UK Cancer Vaccine Launch Pad are expanding access to personalized mRNA cancer‐vaccine trials (not OMV‐based) across tumor types [75]. Collectively, common immune readouts across preclinical/early clinical efforts include rapid DC/NK recruitment, Th1‐skewed responses, and memory CD8^+^ T‐cell expansion capable of rejecting tumor rechallenge [40, 73, 76] (Figure 5).

## 5 Combinatorial Strategies With Bacterial Membrane Vaccines

One of the most promising aspects of bacterial membrane‐based vaccines is their adaptability to multimodal immunotherapy frameworks. Unlike peptide or mRNA vaccines, OMVs and BGs inherently couple antigen scaffolding with PAMPs, enabling simultaneous antigen presentation and DC activation [35, 44]. This duality makes them ideal partners for interventions that relieve inhibitory checkpoints, amplify innate sensing, or remodel the tumor microenvironment.

### 5.1 Checkpoint Blockade Synergy

Immune checkpoint inhibitors (ICIs) such as anti‐PD‐1/PD‐L1 antibodies are most effective in tumors with pre‐existing T‐cell infiltration [77]. OMVs and BGs can overcome this limitation by priming de novo tumor‐specific T cells, thereby supplying the effector pool that ICIs maintain. Recent strategies have integrated immune checkpoint modulation directly into vesicle platforms, including OMV‐based lysosome‐targeting chimeras (LYTACs) designed to promote PD‐L1 degradation [78] and nanobody‐functionalized OMVs that concurrently activate dendritic cells and reprogram suppressive myeloid populations [79]. These constructs exemplify how vesicles function as modular carriers that combine immune activation with checkpoint relief.

### 5.2 Cgas‐STING Pathway Agonists

Combining OMVs with stimulator of interferon genes (STING) agonists is another promising strategy to enhance IFN‐I production and cytotoxic T‐cell priming. While OMVs engage TLR and NOD pathways, STING activation adds complementary cytosolic sensing, generating a broader inflammatory milieu conducive to crosspriming [80]. Vesicular delivery of STING agonists enables cytosolic access in DCs and coordinates multiple PRR pathways within the same APC. Most progress to date has come from engineered whole bacteria, exemplified by SYNB1891 (*E. coli* Nissle), which activates STING in phagocytes and elicits durable antitumor immunity in mice. Applying this strategy to OMV‐based carriers could achieve comparable pathway coordination in a cell‐free format, with greater modularity and safety for vaccine development [81]. The conceptual synergy is particularly compelling for immune‐cold tumors that fail to respond to ICIs alone [31]. However, because bacterial membrane platforms inherently activate innate immune pathways through PAMP‐mediated signaling, future studies should define optimal dosing regimens and therapeutic windows that maximize efficacy while minimizing excessive inflammatory responses.

### 5.3 Phototherapy‐Induced Immunogenic Cell Death

Photothermal and photodynamic therapies (PTT/PDT) can induce immunogenic cell death (ICD), releasing tumor antigens and danger signals. However, without adjuvants, these signals rarely translate into durable systemic immunity [82]. OMVs can address this limitation by providing maturation cues and antigen scaffolds, thereby converting ICD into an in situ vaccination event. Near‐infrared responsive OMVs carrying indocyanine green (ICG) or tumor necrosis factor‐related apoptosis‐inducing ligand have been shown to ablate primary tumors and confer systemic protection against tumor rechallenge, with hybrid OMV‐based phototherapeutic systems increasingly reported as promising approaches to suppress secondary tumor growth [52, 83].

### 5.4 Chemotherapy and Gene‐Silencing Combinations

Bacterial vesicles have been engineered to encapsulate cytotoxic drugs or siRNAs alongside their intrinsic immune ligands. One design codelivered doxorubicin with CD47‐siRNA, simultaneously inducing ICD and silencing the CD47‐SIRPα “don’t‐eat‐me” signal on tumor cells, thereby promoting phagocytosis and crosspresentation [34]. Paclitaxel‐loaded BGs cloaked with tumor membranes further demonstrated how vesicles can transform conventional cytotoxic into immune‐productive interventions by combining homotypic targeting with bacterial adjuvanticity [32].

### 5.5 Tumor‐Membrane Cloaking and Hybrid Vesicles

Fusing bacterial vesicles with tumor‐derived membranes creates carriers that display autologous antigen repertoires while retaining PAMPs. These hybrids achieve both tumor homing via homotypic adhesion and broad antigen presentation, addressing intratumoral heterogeneity [35]. When combined with checkpoint blockade or STING agonists, they form integrated “all‐in‐one” vaccine systems [44].

### 5.6 Radiotherapy and Oncolytic Viruses

Bacteria‐derived vesicles have also been paired with radiotherapy, where they enhance antigen release, promote dendritic‐cell activation, or serve as carriers for radioisotopes [44]. Similarly, bacteria‐virus combinations have been explored in prime‐boost regimens or “boarding” strategies, leveraging bacterial tropism to amplify viral oncolysis [44, 81]. Both approaches extend the reach of vesicle vaccines into new therapeutic niches.

### 5.7 Microbiome Modulation and Small‐Molecule Immunotherapy

Commensal bacteria such as *E. coli* Nissle have been explored as delivery vehicles for checkpoint blockade [84]. Given that TGF‐β inhibition can restore sensitivity to PD‐L1 therapy [85], combining these approaches offers a rational path to enhance ICI responsiveness. Vesicle vaccines thus operate within a broader ecological context where gut and tumor‐resident microbes shape checkpoint efficacy and toxicity [86].

### 5.8 Toward Rational Design of Combinations

The multiplicity of approaches raises critical questions about dosing, sequencing, and safety. For instance, priming with OMVs before ICIs may optimize effector persistence, whereas phototherapy combinations require synchronization with vesicle accumulation. Risks of cytokine storm when layering innate agonists underscore the importance of detoxified strains and lipid‐A engineering [45, 87]. Equally, manufacturing considerations remain unresolved: OMV composition can vary with bacterial growth phase and culture conditions, and protein‐load heterogeneity contributes to batch‐to‐batch variability challenges noted in multiple OMV vaccine reviews [61, 88]. Continuous, detergent‐free production methods and use of well‐characterized chassis such as *Neisseria*‐derived OMVs are being advanced to address these gaps [44]. Biomarker development will be crucial. Indicators such as dendritic‐cell activation (CD80/CD86) [89], IFN‐I signatures [90], T‐cell receptor (TCR) clonality [91], and intratumoral CD8^+^/NK infiltration [52] are likely to guide rational combination strategies and patient selection. Emerging tools such as spatial transcriptomics and imaging mass cytometry will further clarify the immunological niches that OMVs remodel when used in combination settings [53, 92].

**FIGURE 6:**
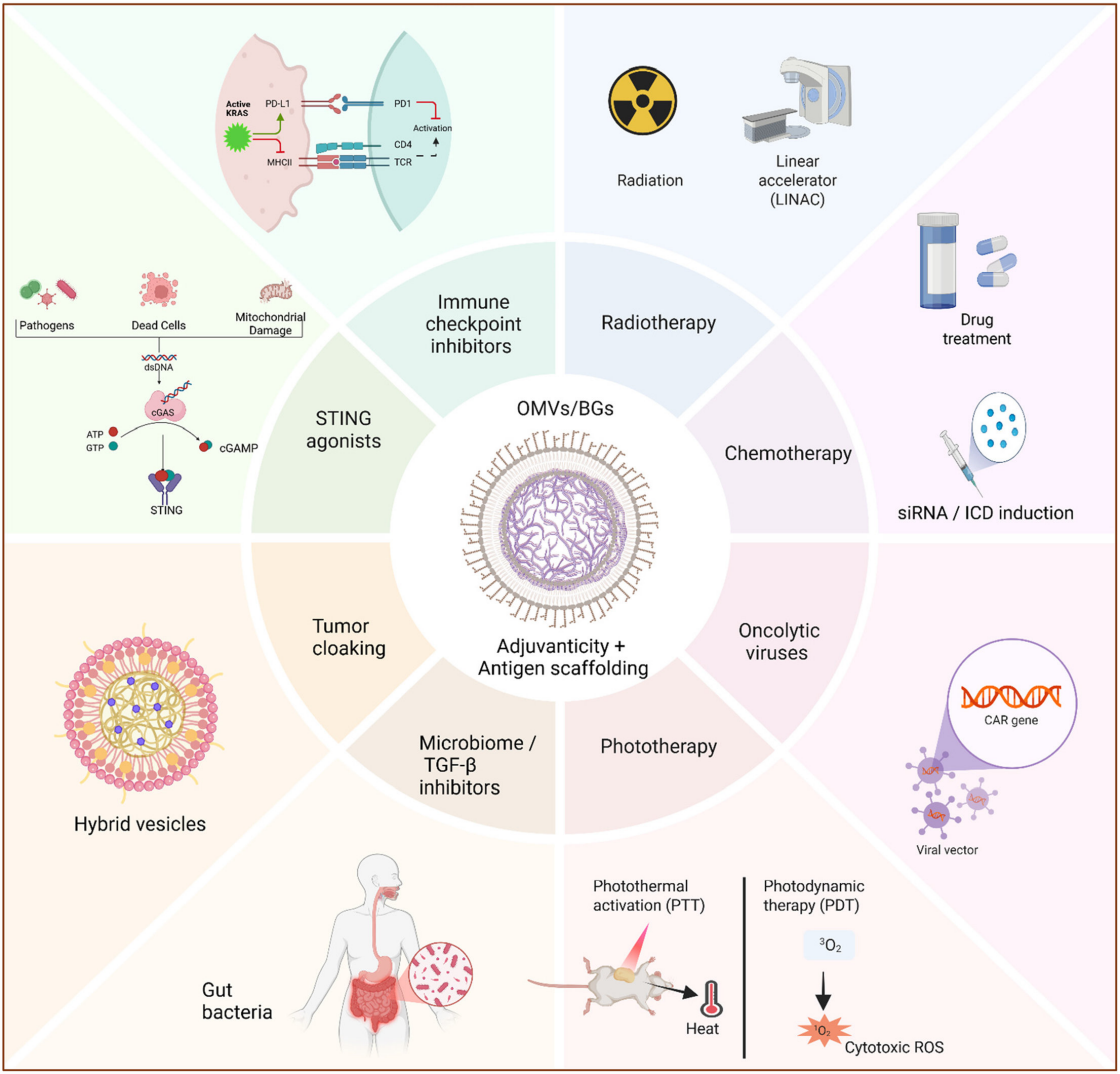
**Combinatorial strategies integrating bacterial membrane vaccines with complementary cancer therapies.** Engineered outer‐membrane vesicles (OMVs) and bacterial ghosts (BGs) function as dual adjuvant‐antigen scaffolds that synergize with immune checkpoint inhibitors, STING agonists, oncolytic viruses, and conventional modalities such as chemotherapy, radiotherapy, and phototherapy. Hybrid vesicles, tumor cloaking, and microbiome modulation further enhance immune activation and tumor targeting, establishing OMVs/BGs as versatile cores in multimodal cancer immunotherapy.

Taken together, bacterial membrane vaccines are not isolated tools but integration platforms. They potentiate ICIs, STING agonists, phototherapy, chemotherapy, radiotherapy, and even viral or microbiome‐based interventions (Figure 6). By embedding adjuvanticity, antigen scaffolding, and modular functionalization, they convert otherwise modest interventions into systemic immunotherapies. Future progress will hinge on rational design guided by emerging biomarkers, including dendritic‐cell activation states, interferon‐driven transcriptional signatures, TCR clonality, and spatial mapping of immune niches [31, 43]. With such integration, bacterial membrane vaccines are poised to become a cornerstone of next‐generation combination cancer immunotherapy.

## 6 Translational Challenges

The development of bacterial membrane‐based cancer vaccines, such as OMVs and BGs, has reached a promising stage in preclinical and early clinical studies. However, their successful translation into widespread clinical use is constrained by complex manufacturing hurdles, regulatory expectations, and ethical considerations. These challenges mirror those faced by mRNA and viral vaccines but are magnified by the unique biological complexity of bacterial systems.

### 6.1 Manufacturing: Good Manufacturing Practice Scale‐up, Endotoxin Control, and Consistency

Manufacturing OMV or BG vaccines at clinical scale requires rigorous adherence to Good Manufacturing Practice (GMP) standards. While laboratory‐scale production typically uses small fermenters and centrifugation, clinical‐grade processes demand large bioreactors, tangential flow filtration, and chromatographic purification [44]. Maintaining sterility and batch‐to‐batch reproducibility in such systems is challenging because vesicle yield and composition are influenced by growth medium, temperature, oxygenation, and bacterial strain background. Even minor deviations can alter lipid, protein, and nucleic acid content, complicating downstream standardization.

Endotoxin control remains the most pressing bottleneck. LPS, particularly its lipid A moiety, provides intrinsic adjuvanticity through TLR4 signaling but also carries the risk of systemic inflammation and septic shock. Regulatory guidelines restrict endotoxin levels in parenteral drugs to below 5 EU/kg body weight: a threshold difficult to achieve with LPS‐rich vesicles. Multiple strategies are being explored, including genetic detoxification via knockout of *lpxM*, *lpxL*, or *pagP* genes to produce under‐acylated lipid A variants with reduced toxicity [45] and the use of engineered *E. coli* chassis such as ClearColi, which generate nontoxic lipid IV‐A while retaining vesicle formation [43]. Downstream purification through chromatographic or detergent‐based methods is also employed, although these often compromise yield and structural integrity [44].

A critical challenge is balancing immunogenicity with safety. Over‐detoxification can blunt vaccine potency, while residual endotoxin poses potential safety risks. Unlike chemically defined mRNA vaccines, OMVs and BGs inherently display biological heterogeneity, making precise dose–response control more difficult. Biodistribution represents an additional translational challenge for OMV‐based therapeutics. Following systemic administration, OMVs are preferentially taken up by cells of the mononuclear phagocyte system and can accumulate in macrophage‐rich organs such as the liver and spleen, potentially limiting the proportion of vesicles reaching tumor sites. While this distribution contributes to immune activation, excessive sequestration may reduce therapeutic efficacy and increase the risk of off‐target inflammatory responses, including systemic cytokine release and hepatic immune activation. To address these limitations, several engineering strategies have been explored, including PEGylation, ligand‐mediated targeting, and tumor membrane cloaking, which can prolong circulation time, improve tumor accumulation, and reduce nonspecific uptake [26]. A better understanding of OMV biodistribution and pharmacokinetics will be essential for optimizing the balance between systemic immune stimulation and tumor‐selective delivery [27].

Another major issue lies in lot‐to‐lot consistency, as vesicle composition can vary not only between production batches but also within a single batch over time. To ensure reproducibility, advanced analytical tools such as proteomic and lipidomic profiling and nanoparticle tracking are increasingly employed to define critical quality attributes (CQAs) [93, 94]. Stability also remains a key limitation: OMVs may aggregate, lose cargo integrity, or undergo bioactivity degradation during storage. Optimization of lyophilization protocols, cryoprotectants, and cold‐chain logistics is therefore essential to preserve vaccine stability and function [93, 95].

### 6.2 Regulatory Expectations and Personalized Vaccines

Regulatory frameworks for therapeutic vaccines are largely shaped by experiences with mRNA (e.g., COVID‐19 vaccines) and viral vectors (e.g., adenoviral platforms). Both systems provide a clear molecular blueprint: defined nucleotide sequences or viral backbones. In contrast, OMVs are complex, multicomponent structures containing proteins, lipids, and nucleic acids. Regulators will require robust demonstration that these components are consistent, safe, and nonpathogenic [95]. Toxicology studies must rigorously characterize cytokine responses, while long‐term safety evaluation will be needed to rule out autoimmunity, chronic inflammation, or microbiome disruption. These requirements are especially stringent in oncology, where patients are often immunocompromised and receive checkpoint inhibitors or chemotherapy, raising the risk of adverse drug‐to‐drug interactions.

A further challenge emerges with patient‐specific OMV vaccines decorated with individualized neoantigens. While analogous in concept to personalized mRNA vaccines, these microbial formulations face the added complexity of biological heterogeneity, with each bespoke batch requiring rapid but comprehensive sterility, endotoxin, and potency assays. Developing adaptive regulatory pathways that accommodate individualized microbial products without paralyzing delays will be essential. Precedents from prophylactic OMV vaccines such as Bexsero and MeNZB demonstrate that vesicle‐based products can meet stringent potency and safety standards, but their application in healthy populations is far less complex than cancer immunotherapy, where immune suppression, heterogeneous disease biology, and combination regimens demand much stricter oversight [95]. Repeated administration may represent a translational challenge for bacterial membrane‐based vaccines. As OMVs contain nonself‐bacterial components, repeated dosing could induce anticarrier immune responses that accelerate vesicle clearance and reduce the efficacy of subsequent administrations, analogous to antivector immunity observed with viral vector vaccines. Further studies are needed to determine the impact of this phenomenon on long‐term therapeutic efficacy [26].

### 6.3 Ethical Considerations: Animal Welfare and Biosafety and Patient Trust

Most OMV and BG vaccines are validated in murine tumor models, which require tumor implantation and repeated sampling. While essential for mechanistic insights, such models raise concerns under the 3Rs principle (Replacement, Reduction, Refinement). The emergence of humanized mice and tumor organoid systems offers alternatives that may reduce reliance on animal models [96]. Ethical approval for preclinical testing is increasingly tied to the demonstration that animal use is minimized and justified by translational relevance.

Even nonreplicating platforms like BGs carry potential biosafety risks. Residual bacterial DNA, toxins, or uncharacterized proteins could trigger off‐target immune responses. Live‐attenuated bacterial vectors, though powerful, risk uncontrolled colonization or horizontal gene transfer. Therefore, GMP production must be conducted under biosafety level 2 or higher, with stringent containment and waste inactivation procedures [44]. Patient‐facing ethical considerations also matter. Informed consent documents must clearly articulate uncertainties around long‐term risks, including microbiome perturbation, systemic inflammation, and repeated dosing effects. Transparent communication of these risks is essential for patient trust, especially in early‐phase oncology trials.

### 6.4 Lessons From Case Studies

**FIGURE 7:**
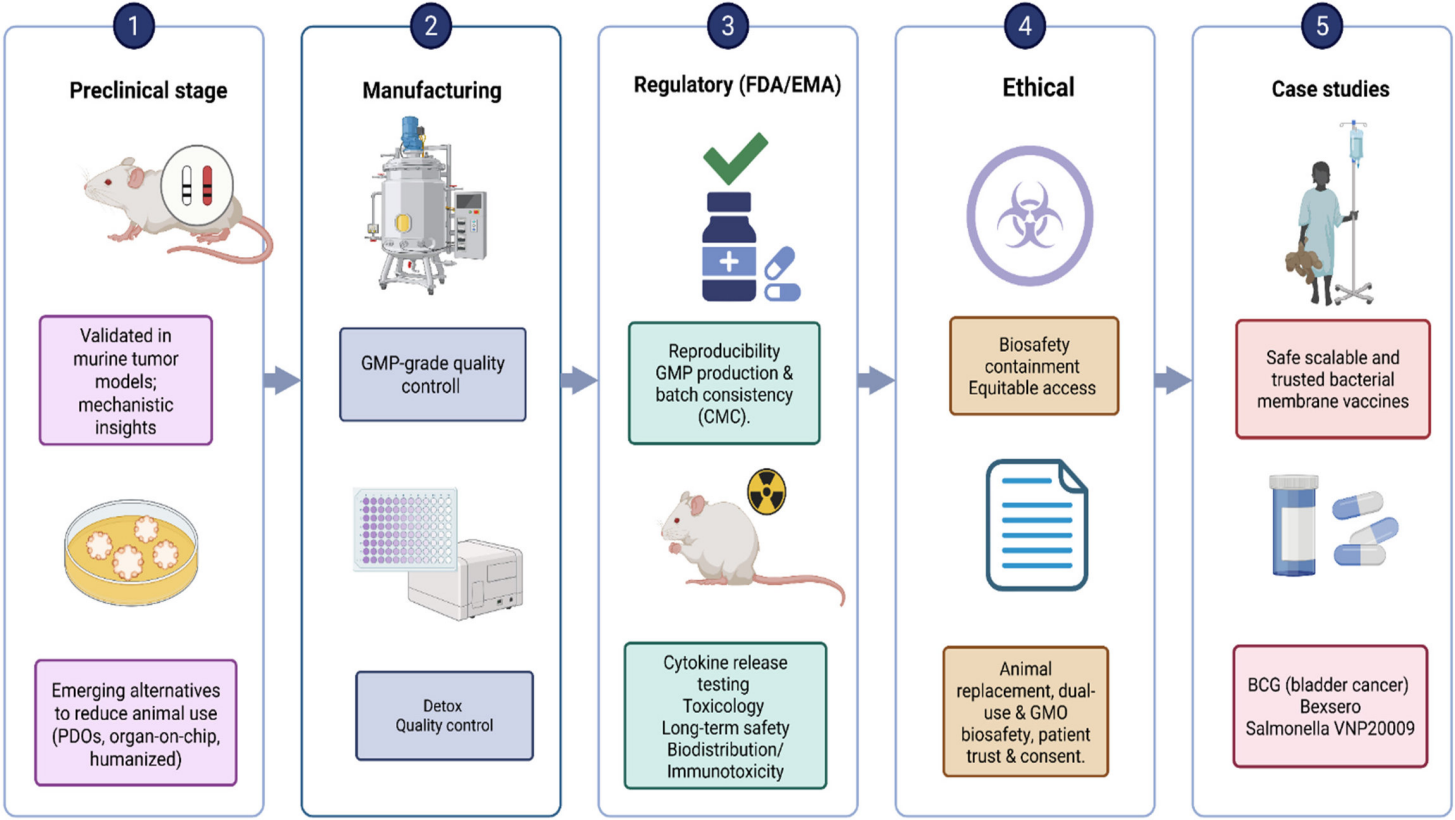
**Translational challenges for bacterial membrane vaccines.** A stepwise overview of the pipeline from preclinical discovery to case studies: (1) Preclinical: validated in murine models with emerging PDOs, organ‐on‐chip, and humanized systems. (2) Manufacturing: scalable bioreactor production with lipid A detox and GMP‐grade QC. (3) Regulatory: FDA/EMA focus on reproducibility, batch consistency, and safety testing. (4) Ethical: biosafety, dual‐use safeguards, equitable access, patient trust, and animal replacement. (5) Case studies: BCG and Bexsero demonstrate success, while Salmonella VNP20009 underscores failure due to toxicity.

Past therapies provide important lessons for bacterial membrane vaccines. Sipuleucel‐T proved individualized immunotherapy is feasible but prohibitively complex and costly. BCG therapy remains a microbial success yet carries severe, unpredictable toxicities. Licensed meningococcal OMV vaccines such as Bexsero and MeNZB demonstrate scalable GMP manufacturing but only in prophylactic settings. Conversely, the attenuated *Salmonella* strain VNP20009 failed in trials due to toxicity and poor tumor colonization, while the granulocyte–macrophage colony‐stimulating factor‐secreting whole‐cell vaccine (GVAX) showed strong immunogenicity but ultimately lacked efficacy in pancreatic cancer. Together, these examples highlight both the promise and pitfalls of translating microbial platforms into oncology. Overcoming these translational barriers will be essential for integrating bacterial membrane vaccines into personalized and scalable cancer immunotherapy pipelines (Figure 7).

## 7 Future Directions

### 7.1 Artificial Intelligence‐Guided Antigen Discovery and Computational Vaccine Design

Challenges in epitope prediction can be overcome by integrating artificial intelligence into vaccine pipelines. Advanced deep learning models now incorporate peptide‐MHC binding, proteasomal cleavage, and TCR recognition into unified predictions, yielding far more accurate forecasts of immunogenicity than motif‐based methods [12, 46, 47]. For OMV platforms, these tools can extend beyond antigen choice to codon optimization and regulatory element design, ensuring efficient vesicle loading. In the long term, coupling AI‐driven discovery with rapid bacterial engineering could enable adaptive vesicles that evolve alongside tumor mutations, with barcoded epitope libraries updated iteratively, analogous to seasonal influenza [12].

### 7.2 CRISPR Chassis Engineering and Synthetic‐Biology Platforms

CRISPR‐based gene editing enables precise control of bacterial genomes to enhance safety, immunogenicity, and functional versatility of vaccine strains. Targeted deletion of virulence genes, secretion regulators, or metabolic pathways allows generation of nonreplicating yet metabolically active chassis suitable for therapeutic use [31, 97]. CRISPR interference (CRISPRi) systems also permit tunable knockdown of essential genes to balance growth and vesiculation rates without permanent mutation [37].

Beyond knockouts, CRISPR‐Cas tools facilitate site‐specific insertion of synthetic operons encoding antigens, secretion tags, or immunomodulatory molecules [8]. These modular circuits enable coordinated expression of multiple payloads under environmental or chemical control, forming the basis for programmable “smart” bacteria that can sense, compute, and respond within the tumor microenvironment [44].

Synthetic‐biology platforms further expand this framework by integrating genetic logic gates, inducible promoters, and biosensors to dynamically regulate antigen production and immune‐stimulating signals [43].

### 7.3 Next‐Generation Hybrid Cloaking Strategies

Future developments may extend current tumor‐membrane cloaking approaches by incorporating alternative mammalian membrane sources and multifunctional designs. For example, dendritic‐cell membranes could enhance antigen presentation and T‐cell priming, whereas erythrocyte‐derived membranes may improve circulation time and reduce rapid clearance. Advances in membrane engineering may also enable programmable hybrid vesicles that combine tumor targeting, immune modulation, and controlled cargo delivery within a single platform. Such next‐generation systems could provide greater control over biodistribution, immunogenicity, and therapeutic efficacy while maintaining favorable safety profiles [35].

### 7.4 Automation and Distributed Biofoundries

Manufacturing complexity can be mitigated by advances in automation. Integrated biofoundry platforms that combine strain engineering, microfluidic vesicle bioreactors, and real‐time analytics are being developed to standardize production and reduce variability [98, 99]. Machine learning‐driven process optimization, already in use for mRNA vaccine manufacturing, is beginning to be applied to microbial vesicle systems [44]. In the future, distributed modular biofoundries could allow personalized OMV vaccine production at regional hubs, echoing the decentralization of CAR‐T therapies.

### 7.5 Systems Immunology and Biomarker Development

Regulatory uncertainty will be addressed by more precise correlates of vaccine potency. Single‐cell RNA sequencing and spatial transcriptomics are revealing how OMVs remodel tumor–immune interactions [66]. Integration of these data with systems immunology models will support rational vesicle design and biomarker discovery. Candidate biomarkers such as polyfunctional CD8^+^ T cells, TCR clonality, and spatial immune architecture are being explored as predictors of durable response [31, 66, 100, 101]. Establishing such standardized metrics will be key for regulatory acceptance and trial harmonization.

### 7.6 Microbiome‐Derived Vesicles

Finally, vesicles from commensal bacteria are emerging as both natural adjuvants and potential therapeutics. Extracellular vesicles from species such as *Akkermansia* or *Bifidobacterium* have shown systemic immunomodulatory effects and synergy with checkpoint blockade in preclinical models [83]. Harnessing or engineering these commensal vesicles may expand the design space of bacterial membrane vaccines beyond traditional pathogens.

**FIGURE 8:**
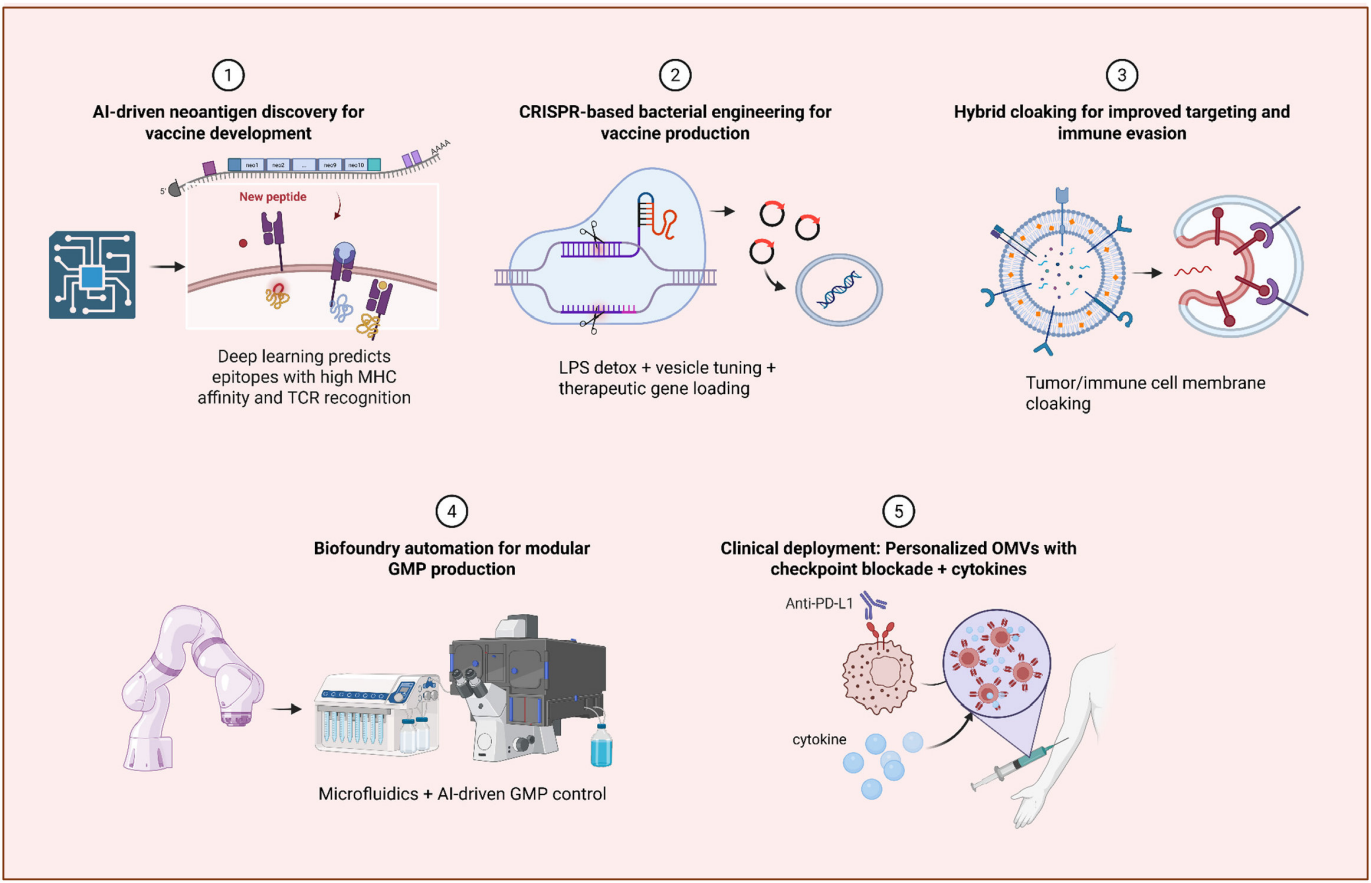
**Vision schematic of next‐generation bacterial membrane vaccine pipeline.** AI‐driven neoantigen discovery enables epitope prediction with high MHC affinity and TCR recognition. CRISPR‐based engineering allows LPS detoxification, vesicle tuning, and therapeutic gene loading. Hybrid cloaking with tumor or immune membranes improves targeting and immune evasion. Biofoundry automation integrates microfluidics and AI‐driven GMP control for scalable production. Clinical deployment envisions personalized OMV vaccines delivered in combination with checkpoint blockade and cytokine therapies.

Taken together, these innovations outline a coherent path forward from the challenges described earlier. AI can refine antigen selection, CRISPR can program vesicles for dual functionality, hybrid cloaking can balance efficacy with safety, and automation can scale production to clinical standards (Figure 8). Systems immunology will provide biomarkers to guide regulation, while microbiome‐derived vesicles broaden the biological palette. If these solutions can be integrated into cohesive pipelines, bacterial membrane platforms may transition from experimental tools into a central pillar of precision cancer immunotherapy.

## 8 Conclusion

Bacterial membranes, encompassing OMVs, BGs, and engineered membrane fragments, have emerged as versatile dual‐function platforms that integrate antigen presentation with intrinsic adjuvanticity. Unlike conventional peptide, mRNA, or viral vaccines, they package both pathogen‐associated molecular patterns and antigenic scaffolds into a single system, driving potent crosspriming and durable T‐cell memory. Advances in genetic fusion systems, conjugation chemistries, lipid A detoxification, and hybrid cloaking now enable precise engineering, while preclinical studies across melanoma, lung cancer, and glioblastoma highlight their capacity to remodel the tumor microenvironment and synergize with checkpoint blockade, chemotherapy, and phototherapy.

Yet clinical translation will hinge on three priorities: standardized potency assays to compare responses across studies, multicenter Phase II trials to move beyond single‐institution pilots, and robust biosafety frameworks to address endotoxin heterogeneity and batch variability. Integration of computational design, CRISPR‐enabled chassis optimization, and automated biofoundry manufacturing will be essential to bridge laboratory innovation with regulatory scalability and clinical deployment. Looking forward, AI‐driven neoantigen prediction, CRISPR‐based membrane editing, and automated biofoundries may overcome these hurdles. As biomarker‐guided immunotherapy advances, bacterial membrane vaccines could provide personalized, modular platforms that unite evolutionary immunogenicity with synthetic programmability, positioning them as a durable pillar of next‐generation cancer immunotherapy.

### 8.1 Final Remarks

Bacterial membrane systems unify antigen presentation and innate immune activation within a single programmable scaffold. Their capacity to reshape the tumor microenvironment, drive durable CD8^+^ responses, and integrate with checkpoint blockade positions them as leading candidates for next‐generation cancer immunotherapy. Advancing to clinical translation will require standardized potency assays, GMP‐grade consistency, and computationally guided antigen design, but current evidence strongly supports their emerging therapeutic relevance.

## Author Contributions

**Hafiza Aasia Malik:** conceptualization; investigation; data curation; methodology; visualization; writing original draft; writing review & editing; project administration. **Urooj Yousaf Virk:** Visualization; writing review & editing. **Mohamed Salah Attia:** data curation; writing review & editing. **Ming Wei:** supervision; & validation. **Jenny Wilson:** supervision; & validation. All authors reviewed and approved the final manuscript.

## Funding

Open access publishing facilitated by Griffith University, as part of the Wiley ‐ Griffith University agreement via the Council of Australasian University Librarians).

## Data Availability

No new data were generated or analyzed in this study. Data sharing is not applicable to this article.
